# Type 1 Diabetes Mellitus in Saudi Arabia: A Soaring Epidemic

**DOI:** 10.1155/2018/9408370

**Published:** 2018-05-08

**Authors:** Asirvatham Alwin Robert, Abdulrahman Al-Dawish, Muhammad Mujammami, Mohamed Abdulaziz Al Dawish

**Affiliations:** ^1^Department of Endocrinology and Diabetes, Diabetes Treatment Center, Prince Sultan Military Medical City, Riyadh, Saudi Arabia; ^2^Department of Dentistry, Prince Sultan Military Medical City, Riyadh, Saudi Arabia; ^3^Division of Endocrinology and Metabolism, Department of Medicine, King Saud University, Riyadh, Saudi Arabia

## Abstract

Type 1 diabetes mellitus (T1DM) is quite prevalent in the world, with a proportion of 1 in every 300 persons and steadily rising frequency of incidence of about 3% every year. More alarmingly, the incidence of T1DM among infants is also increasing, with children as young as 6 months succumbing to it, instead of that at a rather established vulnerable age of around seven and near puberty, when the hormones antagonize the action of insulin. These reports pose a unique challenge of developing efficient T1DM management system for the young children. The Kingdom of Saudi Arabia (KSA) is the largest country in the Middle East that occupies approximately four-fifths of the Arabian Peninsula supporting a population of more than 33.3 million people, of whom 26% are under the age of 14 years. As per the Diabetes Atlas (8th edition), 35,000 children and adolescents in Saudi Arabia suffer from T1DM, which makes Saudi Arabia rank the 8th in terms of numbers of TIDM patients and 4th country in the world in terms of the incidence rate (33.5 per 100,000 individuals) of TIDM. However, in comparison with that in the developed countries, the number of research interventions on the prevalence, incidence, and the sociodemographic aspects of T1DM is woefully inadequate. In this review we discuss different aspects of T1DM in Saudi Arabia drawing on the published literature currently available.

## 1. Introduction

Type 1 diabetes mellitus (T1DM) is one of the most common endocrine metabolic disorders affecting children and adolescence across the world; T1DM is often accompanied by serious acute and chronic complications. Moreover, autoimmune diabetes, which is a chronic disease characterized by insulin deficiency due to pancreatic *β*-cell loss, is known to lead to hyperglycemia [1–4]. Symptomatic onset usually occurs in the childhood or adolescence, although the symptoms sometimes develop much later in life [5]. A recent study stated that the life expectancy of people with T1DM is still approximately 12 years less on average than the general population [6]. As the causes and risk factors associated with T1DM remain not fully understood, the cure and prevention strategies developed until date have been unsuccessful, with most patients depending on a life-long insulin injection treatment [7]. Recently, novel approaches of insulin treatment such as the use of insulin pumps, continuous glucose monitoring, and hybrid closed-loop systems have been proposed [4, 6]. Although intensive glycemic control can reduce the incidence of microvascular and macrovascular complications, most patients with T1DM develop these complications [4]. The Kingdom of Saudi Arabia (KSA), which is the largest country in the Middle East that houses approximately four-fifths of the Arabian Peninsula, supports a population of more than 33.3 million people, of whom 26% are aged < 14 years [8]. Studies indicate, in the recent decades, a significant increase in prevalence and incidence rates of T1DM in Saudi Arabia, mainly among the children and adolescents [9, 10]. However, in comparison with that in the developed countries, the amount of researches conducted in Saudi Arabia on the incidence, prevalence, and sociodemographic properties of T1DM is highly inadequate [10]. In this review we discuss different aspects of T1DM in Saudi Arabia drawing on the published literature currently available.

## 2. Methods

The archives of the PubMed, Scopus, Google Scholar, and Google were searched for relevant literature. The review also included reports from the World Health Organization (WHO) and IDF. The articles were selected by reviewing their titles and abstracts as well as from the bibliography of the selected articles.

Key words used to search for relevant articles included type 1 diabetes, diabetes mellitus, prevalence, incidence, diabetes complication, quality of life, awareness, and Saudi Arabia. These terms were used individually and together to ensure an extensive literature search.

The Critical Appraisal Skills Programme (CASP) guideline was used for case-control and cohort studies as a framework for quality assessment. Studies with major limitations in methods or reporting were excluded [11]. Data concerning sample size, duration, design, and method of the study were extracted and used as indicators of quality examination.

## 3. Global Prevalence and Incidence

The epidemiological data indicate an increase of about 3-4%/year incidence in T1DM globally, with the age of onset younger than ever before [12]. These observations were specific to developed and developing countries, especially the United States of America, Latin America, Europe, Australia, India, Southeast Asia, and China [12–20]. However, the incidence of childhood-onset T1DM varies among countries. For instance, East Asia and native America report the lowest incidences of approximately 0.1–8/100,000/year, while the highest rates have been reported for Finland with >60/100,000/year, Sardinia with 40/100,000/year, and Sweden with 47/100,000/year [12]. Countries with the highest estimated numbers of new cases reported annually include the United States (*n* = 14,700), followed by India (*n* = 11,300) and Brazil (*n* = 7,600) [21]. The prevalence estimates indicated almost 586,000 children aged under 15 years with T1DM across the globe, with the largest proportion in Europe and North America [21, 22]. As compared with the prevalence estimated in previous editions of the IDF, Diabetes Atlas, the proportions have increased in most of the IDF regions, which reflects the well-documented increase in the incidence rate in several countries [21–23]. Most recently, the 8th edition (2017) of the IDF Diabetes Atlas reported that the number of young people (<20 years) living with T1DM worldwide is estimated to be 1,106,500 million [21], which is double the number cited in the previous Diabetes Atlas (2015) [24]. Besides, more than 96,000 children and adolescents under 15 years are estimated to be diagnosed with T1DM annually and when the age range covers up to 20 years, the number is estimated to be more than 132,600 [21].

The prevalence of T1DM has been reported to vary greatly among different countries, within countries, and among different ethnic populations [23]. The global variation in the incidence of T1DM was evaluated by grouping the populations as having “extremely low” (<1/100.000/year), low (1–4/100.000/year), intermediate (5–9.99/100.000/year), high (10–19.99/100.000/year), and extremely high (>20/100.000/year) incidence. The difference in the annual incidence rates of T1DM was compared among different countries of the world (ranging from 0.1 to 57.6 per 100,000 individuals) [25]. The highest incidence was observed in the Scandinavian countries, with the highest report for Finland. A linear relation was noted with a decrease in the incidence rate with decreasing distance to the equator [26]. However, some countries such as Puerto Rico, Kuwait, and Sardinia have an unexplained high increase in the incidence rate [26].

## 4. Situation in Middle East and North Africa

As per the Diabetes Atlas (8th edition) among the Middle East and North Africa (MENA) countries, only Kuwait had a countrywide study conducted within the last five years. Algeria, Jordan, Oman, Pakistan, Palestine, Sudan, United Arab Emirates, and Saudi Arabia had estimates partially based on oral glucose tolerance tests. The prevalence of diabetes statistics for the remaining countries may be underestimated [21].

In the Middle Eastern countries, the incidence rate was 2.5 per 100,000 people in Oman and 22.3 per 100,000 people in Kuwait. Saudi Arabia had the highest rate of children affected with T1DM which accounts for approximately one-quarter of those in the Middle East and North Africa (MENA). This number is comparable to that from Norway and other Scandinavian countries, where T1DM is historically and genetically more prevalent than that in the Middle East, Asia, and Africa [14, 27]. Recently, a study reported that the prevalence and incidence of T1DM were found to be variable among the Arabs, with the existence of only a few national/regional diabetes registries available to support diabetes research, provide reliable data, and help cope with the widespread threat of this disease. Hence, there is a need for establishing a population-based Arab diabetes registry [28].

## 5. Rising Trend in Saudi Arabia

The incidence rate of T1DM has grown in Saudi Arabia over the last 3 decades [29]. However, the data on the incidence/prevalence of T1DM remains limited and only a few systematic reviews have been conducted on the epidemiology of diabetes in this region [30, 31].

**Figure 1:**
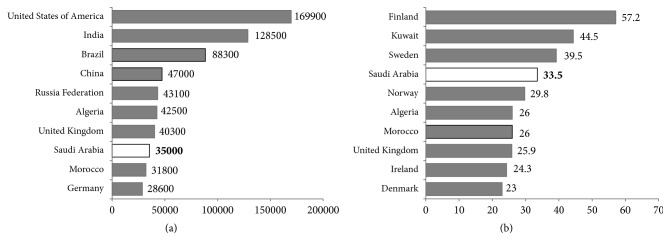
(a) Top ten countries/territories for number of children and adolescents with type 1 diabetes (<20 years). (b) Top ten countries/territories for the incidence rates of type 1 diabetes (<20 years) per 100,000 children per year, 2017 [21].

A study from the Eastern region of Saudi Arabia conducted in 1990–2007 reported the incidence rate of T1DM (aged 0–14 years) has doubled among children in less than two decades from 18.05 per 100,000 children between 1990 and 1998 to 36.99 per 100,000 children between 1999 and 2007, indicating an average annual increase in the incidence of 16.8% [20]. Other studies reported that the incidence of T1DM in Saudi children has been reported to be 27.5 per 100,000 [32] and 29 per 100,000 [9], which are quite high as compared with that in several other countries. In addition, a study reported that Saudi Arabia has some of the world's highest annual incidence rates of T1DM among children (31.4 new cases per 100,000 individuals) [24]. The IDF (2015) reported that Saudi Arabia has 16,100 children (0–14 years) with T1DM; this figure is by far the highest in Saudi Arabia, accounting for over a quarter of the 60,700 total affected individuals in MENA region [24]. However, most recent (2017) report from IDF showed that Saudi Arabia (35,000) has highest number of people with T1DM (0–19 years) and has highest number of new cases (3,900) of T1DM (Figure 1) [21]. Official registration of T1DM cases is insufficient in Saudi Arabia; however, several healthcare practitioners and researchers also argue that the incidence of this disease has risen sharply [33, 34]. Alarmingly, the incidence of this disease among infants is growing, with children as young as six months getting affected, instead of that at the peak age of around seven and at puberty, when the hormones antagonize the action of insulin. A countrywide Saudi Arabian project conducted in 2001–2007 reported that, for 11,874 out of 14,000 selected households and 45,682 children and adolescents surveyed, the study prevalence rate identified was 109.5 per 100,000 individuals. The distribution of T1DM prevalence by region showed that it was highest in the central region (126 cases per 100,000; mostly urban) and lowest in the Eastern region (48 cases per 100,000; mostly rural) of Saudi Arabia [34]. Al-Rubeaan, 2015, also reported that the majority (77.2%) of cases of T1DM were documented in urban rather than rural areas (22.7%) [35].

## 6. Possible Causes and Lack of Research

The onset of T1DM is influenced by multiple genetic and environmental risk factors [36]. As the genetic background of T1DM is extremely complex and the risk of developing T1DM is increased mainly in persons with either HLA-DR3-DQ2 or HLA-DR4-DQ8 haplotypes, however, it is difficult to explain the involvement of HLA alone [37, 38]. On the other hand, the varying viral and nutritional factors across countries may contribute to the etiology of T1DM [39]. The increasing incidence of this disease in the Saudi Arabia can be attributed generally to the rapid lifestyle changes such as change in nutrition, changes in breastfeeding practices, exposure to different environmental pollutants and toxins, and autoimmune deficiency developed as a result of greater hygienic standards and low vitamin D levels, which is shockingly predominant in Saudi Arabia despite the abundance of sunlight in the day time [40–42]. Further, urbanization, misclassification of patients diabetes [9], limited initiatives for prevention and control, and ineffectiveness in creating awareness are also the major reasons for the rising prevalence/incidence of T1DM [10].

Several studies identified that the greater practice of consanguinity, endogamy, and first-cousin marriage in Saudi Arabia is significantly responsible for the creation of several inbreeding communities that have in turn led to an increase in homozygosity of both the HLA haplotypes and non-HLA genes associated with either the protection or the susceptibility to T1DM among the Saudi population [28, 43, 44]. Apart from genetic factors, gender and age have also contributed to the development of T1DM. In general, the incidence rate of T1DM increases from birth and peaks between 10 and 14 years of age, that is, at the puberty [45, 46]. A study from Al-Madinah in the North West Saudi Arabia between 2004 and 2009 also reported a significantly higher incidence in the 10–12-year age group than in younger children [9]. Further they reported a significantly higher incidence of T1DM in girls than in boys (33.0 versus 22.2 per 100,000 children) [9]. Another study also reported that the incidence of T1DM was significantly higher among females (31.1 cases per 100,000) than males (24 cases per 100,000) [32]. In females the highest incidence rate was reported for those aged 7–11 years and for males similar rates were reported for those aged 8–12 years [32].

The major reasons for lack of medical research in Saudi Arabia may due to lack of research training, time, lack of supervisors, and involvement and students are not aware of the usefulness and importance of scientific research [47, 48].

## 7. Complications

### 7.1. Micro- and Macrovascular Complications

Intensive glycemic control has been demonstrated to reduce the long-term vascular complications of hyperglycemia in T1DM. Unfortunately, diabetic complications continue to be a major cause of morbidity and mortality in patients with T1DM and mainly with cardiovascular disease (CVD) [49]. A study from Jeddah in Saudi Arabia reported that (in an age group of 5–70 years), microvascular complications occur in 24%, retinopathy in 7%, nephropathy in 2%, and neuropathy in 6% of T1DM patients. More than one microvascular complication occurred in 9%, macrovascular complication in 6%, cardiovascular complication in 4%, cerebrovascular disease in 1%, and peripheral vascular complication in another 1% of the patients. Furthermore, 30% of the patients had both micro- and macrovascular complications [50]. Another study (included 228 T1DM children and adolescents) from Jeddah, Saudi Arabia, reported that acute complications of ketoacidosis occur in 65.4% of patients and hypoglycemic attacks in 68.9%. Long-term complications were found as follows: retinopathy (4.4%), microalbuminuria (16.2%), and dyslipidemia (8.3%) [51].

### 7.2. Diabetic Ketoacidosis

Diabetic ketoacidosis (DKA) is a major life threatening recurrent complication of T1DM and it is the most common reason of death in children and adolescents with T1DM [52]. In some cases, DKA may be the first sign of previously undiagnosed diabetes, but it may frequently happen in those who previously have diabetes [53].

Worldwide, more than 96,000 children and adolescents aged under 15 years develop T1DM annually [21], and 13% to 80% of these children present with DKA at the time of diagnosis [54]. The highest frequencies for DKA at presentation of T1DM are seen in Saudi Arabia (44.9%) [55] and the United Arab Emirates (80%) [56]. The lowest frequencies for DKA at presentation of T1DM are found in Hungary (23%), Finland (22%), Canada (18.6%), and Sweden (14%) [54]. A systematic review (65 studies) comprising over 29,000 children from 31 countries reported that the incidence of DKA varied sixfold, from 80% in the United Arab Emirates to 12.8% in Sweden. The study also demonstrated that the highest incidences were seen as 59% in Saudi Arabia [54], 80% in United Arab Emirates, 67% in Romania, 65% in Taiwan, and the lowest incidences were seen in Sweden (14%), Canada (18.6%), Finland (22%), and Hungary (23%) [54]. An increased risk of DKA may be due to younger age, diagnostic error, lower body mass index, ethnic minority status, earlier infection, lack of health insurance, and delayed treatment [57].

A retrospective study (children aged < 12 years) from Tabuk, Saudi Arabia, reported that 106 of the 279 children (38.0%) had presented with DKA. Further they described that female patients, underweight children, and those aged 0–3 years established the highest risk of developing DKA [58]. Recently, a cross-sectional study conducted among 103 T1DM adolescents at central region of Saudi Arabia reported that the frequency of recurrent diabetic ketoacidosis (RDKA) was significantly greater in the T1DM adolescents with a higher HbA1c level and lipodystrophy and those who had dropped insulin treatment. It was further suggested that comprehensive multidisciplinary diabetes education should be offered to minimize the modifiable risk factors of Saudi T1DM patients [53].

### 7.3. Peripheral Neuropathy

The diabetic peripheral neuropathy (PN) is a major long-term complication in children and adolescents with T1DM, with significant morbidity and mortality [59, 60]. Currently there are only limited studies available in Saudi Arabia on peripheral neuropathy among T1DM patients and a study from Saudi Arabia reported that 6% of patients with T1DM have PN. Recently, a meta-analysis performed on studies representing different Arab countries with a total number of 2243 T1DM patients reported that PN is common in adults and children with T1DM, but prevalence differs extensively. Various studies revealed significant differences in the prevalence of PN among Arabs, ranging from low (0–0.5%) as in Libya to very high (44–71%) as in Jordan and Egypt [61]. They further reported the overall prevalence of PN among T1DM patients in the Arab region as 18% [61]. The dissimilarity of PN among the different Arab countries possibly reflects the genetic and socioeconomic heterogeneity of the people in this Arab region. Further, differences in the methods of measuring the outcome between different studies could also associated with this heterogeneity [62].

### 7.4. Vitamin D Deficiency

Studies suggest an association between vitamin D deficiency in early life and the later onset of T1DM. However, data from Saudi Arabia are limited [20, 63]. In a prospective cross-sectional study conducted in Saudi Arabia, the measured serum 25-hydroxy vitamin D (25OHD) level was mild in 64% of the children, moderate in 16%, and severe in 4% as compared with 52%, 6%, and 1%, respectively, in the normal children. They further stated that overall 84% of the T1DM children and 59% of the healthy ones were found to be vitamin D deficient [64]. Another recent study from central region of Saudi Arabia stated that the mean levels of 25OHD were significantly lower in patients with T1DM than the normal adults. The study further identified that 66.7% of patients with T1DM mildly, 31.7% moderately, and 3.3% severely had vitamin D deficiency as compared with 41.7% mildly, 31.7% moderately, and 5% severely deficient cases in the normal adults [65]. A study from western region of Saudi Arabia found that 70.2% of the patients had vitamin D deficiency; they also reported that various other factors apart from T1DM might contribute to vitamin D deficiency [51] which is in agreement with the latest study from UK, which reported that low 25OHD level is prevalent among children and adolescents with T1DM [66].

### 7.5. Celiac Disease

The association between T1DM and celiac disease (CD) was expected, as both the conditions include an increase in the frequency of human leukocyte antigen- (HLA-) DR3 and other HLA [67–69]. However, in most patients, it is often found that CD is diagnosed shortly or sometimes some year after the onset of T1DM [70]. The prevalence of CD in T1DM is considerably high, with a prevalence rate 5–7 times more than that in nondiabetic patients and an estimated prevalence of 4.4–11.1% in different populations (for general population 0.5%) [71].

A meta-analysis review of data for 26,605 patients projected a worldwide prevalence of biopsy proven celiac disease among T1DM to be 6% [72]. However, an extensive variation was noted as in France it was 1.6%, in USA 4.6–7.0%, in Italy 3.6–6.6%, in Sweden 9-9.7%, and in the United Kingdom 3.3–4.0% [72]. This variation could be attributed to the duration of the disease and the age at diagnosis, in addition to genetic susceptibility [73]. In Saudi Arabia a study examined 218 adults with T1DM at a large center in Saudi Arabia and found a 7.3% prevalence of celiac disease in them [74]. Other studies also reported a prevalence of celiac disease in patients with T1DM in Saudi Arabia to be 4.8–11.3% [75]. Studies conducted from different regions of Saudi Arabia reported that in southern region 10.4%, in central region 11.3%, and in western region 11.2% of patients with T1DM possess celiac disease [67, 69, 75]. It is important to note that a recent study finding shows a high prevalence of celiac disease (19.7%) among TIDM patients in Saudi Arabia [51].

### 7.6. Autoimmune Thyroiditis

The associations between subclinical autoimmune thyroiditis and T1DM have been frequently studied [76]; however, their role in the glycemic state has not been well investigated. Nevertheless, extremely few data are available from Saudi Arabia about this despite an increase in the incidence of this disease. A study on 132 Saudi children showed a higher distribution of anti-glutamic acid decarboxylase (GAD), thyroid autoantibodies antithyroid peroxidase (TPO), and anti-thyroglobulin (TG) among T1DM patients (56.8, 36.4, and 19.7%, resp.) in contrast to a lower distribution among controls (5.6, 9.7, and 4.2%, resp.) [77]. Furthermore, a group of patients with positive autoimmune thyroid antibodies were associated with a significant increase in the hemoglobin A1c (HbA1c) level in comparison with that in the other patients' group with negative thyroid antibodies. On the other hand, the level of TSH was quite high (approximately 9.8%) in diabetic patients, while it was only 1.4% among the healthy individuals. Another study on 305 children and adolescents with T1DM reported a significant increase in the risk of developing thyroid dysfunction with a high prevalence of 21.3% [78]. Recently, Al-Agha et al. reported thyroid dysfunctions due to autoimmune thyroiditis were found in 4.8% of the patients, as well as subclinical hypothyroidism in 9.4% of the patients [51].

## 8. Quality of Life

Measurement of the quality of life (QoL) varies in specific dimensions but comprises aspects of physical, emotional, and social well-being [79]. It is well known that children with T1DM have to deal with a multifaceted and demanding daily treatment regime which can have a negative impact on the QoL of these patients [80]. According to few Saudi studies, they have been found to have more psychological problems with significant increase of risk of depression mainly among the female gender [81, 82]. However, in Saudi Arabia less emphasis is placed on addressing the psychosocial components of disease management and the impact it has on adolescents' QoL.

A cross-sectional study of mothers of T1DM children aged 6–12 years reported a declining trend of school cooperation, psychosocial status, activity level, medical and emergency care, aspects of child education, diet, and organizational and social support [83]. Another study conducted among 214 adolescents (13–18 years) with T1DM at the central region of Saudi Arabia reported that female gender, multiple daily injections, longer duration of T1DM, diabetic ketoacidosis, and adolescents with high HbA1c level had poor QoL outcome [81]. Recently, a cross-sectional study on 315 patients with T1DM (aged 12–18 years) and their caregivers reported a poor quality of life. Female gender and late adolescent age were found to be predictors of lower QoL outcome for adolescents with T1DM [82]. Majority of the studies from Saudi Arabia reported that female patients with T1DM have poor QoL [81, 82]. In addition to poor QoL, research from Saudi Arabia reported that more than one-fourth of female with T1DM experiencing menstrual irregularity and a delay in the age of menarche [84].

## 9. Awareness

Foremost among the challenges of T1DM is the lack of awareness about it among the general public, T1DM patients, and parents of T1DM children. It is crucial to improve this situation in order to effectively counter the problem of T1DM [85]. The disease, when left unmanaged, poses various challenges to the patient and healthcare providers in terms of the development of diabetic complications, thereby decreasing the life expectancy of the affected children [33, 85]. A recent study from Saudi Arabia reported that only 31.2% of children and adolescents with T1DM are well controlled which clearly demonstrated low awareness about the diabetes control among the T1DM patients [86]. Another study from Saudi Arabia reported that more than 90% of the children and adolescents with diabetes were unaware of their disease; the proportion of patients with type 1 and type 2 diabetes who were aware of their disease accounted for a mere 0.45%, while the newly identified cases with diabetes and impaired fasting glycaemia accounted for 10.4% [35]. Other studies also reported only limited awareness of diabetes among children and young adult population in Saudi Arabia [35, 55, 87].

## 10. Possible Solutions

There is no effective intervention presently existing to prevent T1DM and the prevention of loss of *β* cells in T1DM is a major target of current research. However, with the provision of an uninterrupted supply of insulin and blood glucose testing equipment, combined with a healthy lifestyle, people with T1DM can live healthy and satisfying lives. In countries where there is inadequate access to insulin and insufficient health service provision, children and adolescents with T1DM suffer dreadful complications and early mortality [21].

In Saudi Arabia, the Ministry of Health not only provides treatment to all patients with diabetes but also makes the effort to offer defensive measures and mass education. However, there is still much to accomplish [10, 33, 88]. In order to prevent or delay the progress of complications of T1DM, it is necessary to train and recruit professionals so as to bring the staff complement for the pediatric diabetes facility in line with internationally accepted levels. The diabetes community of scientists, clinical trialists, patients, families, funding agencies, education institutions, and regulatory agencies of Saudi Arabia must work together in a cooperative and collegial manner to prevent or delay the development of complications of T1DM [89]. Further, it is essential to raise awareness on the importance of a healthy diet and physical activity, especially among children and adolescents, and incorporate healthy environments into urban planning.

It is important to note that most of the published researches in Saudi Arabia are cross-sectional with small sample sizes, dealing with single center and single city/region of the country. Therefore, comprehensive studies and innovative research are urgently needed and the clinical trials must be well designed, adequately powered, carefully controlled, cautiously conducted, and multicenter approaches in order to prevent or delay the development of the devastating microvascular and macrovascular complications of the T1DM in Saudi Arabia.

## 11. Conclusion

There is a dearth of meticulously conducted research on T1DM in Saudi Arabia. Considering the increasing prevalence of T1DM in Saudi Arabia, especially in infants and young children, the research interventions need to be significantly improved. Moreover, it is critical to develop appropriate management programs for controlling T1DM and allocating health resources appropriately for this traumatic condition. Research efforts should focus on achieving early diagnosis, preventing *β*-cell loss, and developing better treatment options to improve the quality of life and prognosis of the affected individuals.

## Additional Points

*Data Sharing Statement*. There is no data sharing as this manuscript and the data were not published elsewhere.
